# Does Inhalation of Virgin Coconut Oil Accelerate Reversal of Airway Remodelling in an Allergic Model of Asthma?

**DOI:** 10.1155/2017/8741851

**Published:** 2017-06-04

**Authors:** N. A. Kamalaldin, S. A. Sulaiman, M. R. Yusop, B. Yahaya

**Affiliations:** ^1^Regenerative Medicine Cluster, Advanced Medical and Dental Institute, Universiti Sains Malaysia, 13200 Kepala Batas, Penang, Malaysia; ^2^Department of Pharmacology, School of Medical Sciences, Universiti Sains Malaysia, 16150 Kubang Kerian, Kelantan, Malaysia; ^3^School of Chemical Science and Food Technology, Faculty of Science and Technology, Universiti Kebangsaan Malaysia, 43600 Bangi, Selangor, Malaysia

## Abstract

Many studies have been done to evaluate the effect of various natural products in controlling asthma symptoms. Virgin coconut oil (VCO) is known to contain active compounds that have beneficial effects on human health and diseases. The objective of this study was to evaluate the effect of VCO inhalation on airway remodelling in a rabbit model of allergic asthma. The effects of VCO inhalation on infiltration of airway inflammatory cells, airway structures, goblet cell hyperplasia, and cell proliferation following ovalbumin induction were evaluated. Allergic asthma was induced by a combination of ovalbumin and alum injection and/or followed by ovalbumin inhalation. The effect of VCO inhalation was then evaluated via the rescue or the preventive route. Percentage of inflammatory cells infiltration, thickness of epithelium and mucosa regions, and the numbers of goblet and proliferative cells were reduced in the rescue group but not in preventive group. Analysis using a gas chromatography-mass spectrometry found that lauric acid and capric acid were among the most abundant fatty acids present in the sample. Significant improvement was observed in rescue route in alleviating the asthma symptoms, which indicates the VCO was able to relieve asthma-related symptoms more than preventing the onset of asthma.

## 1. Introduction

Asthma is an airway disorder that is characterised by a specific pattern of inflammation that is largely driven by immunoglobulin E-dependent mechanisms in the airway [1]. Asthma onset can be triggered by internal and external factors, including environmental allergens, stress, cigarette smoke, and changes in weather [2, 3]. In airway remodelling of asthma, a number of changes occur, including epithelial goblet cell hyperplasia and metaplasia, collagen deposition and thickening of the lamina reticularis, smooth muscle hyperplasia, and proliferation of airway blood vessels [4].

Many researchers have studied the antiasthmatic and anti-inflammatory effects of several plant-based therapies, including thymoquinone from the seed of* Nigella sativa*, and the crepe myrtle (*Lagerstroemia indica*) on animal models of chronic and acute airway inflammation [5–9]. Yang et al. (2008) studied the effect of an extract of* Duchesnea chrysantha* on leukocyte infiltration and mucous secretion and reported that the extract reduced inflammation due to leukocyte infiltration and mucous hyperproduction by goblet cells [10].

VCO has been studied extensively because the free fatty acids in this oil have beneficial effects on many aspects of human health and disease such as antibacterial, antiviral, anti-HIV, anti-inflammatory, and antidiabetic properties [11–13]. Other than that, anticancer properties of the VCO have been reported by Kamalaldin et al. (2015) where the VCO was able to induce apoptosis on lung cancer cell and gave no toxic effect towards normal fibroblast cell [14]. The VCO is extensively used as a topical application to treat skin disorders [15]. The efficacy and safety of VCO in reducing visceral adiposity indicate that VCO is efficacious in weight reduction and is safe for human consumption [16]. To provide scientific-based evidence to support anecdotal claims, this study was designed to evaluate the effect of VCO inhalation on airway remodelling in a rabbit model of allergic asthma.

## 2. Materials and Methods

All chemicals used in this study were of analytical grade and were purchased from Sigma-Aldrich (Munich, Germany) unless stated otherwise.

### 2.1. Virgin Coconut Oil

The VCO sample was obtained from Nutrifera (Kelantan Biotech Corp Sdn. Bhd., Kota Bharu, Malaysia).

### 2.2. Development of the Chronic Lung Injury Model

**Figure 1:**
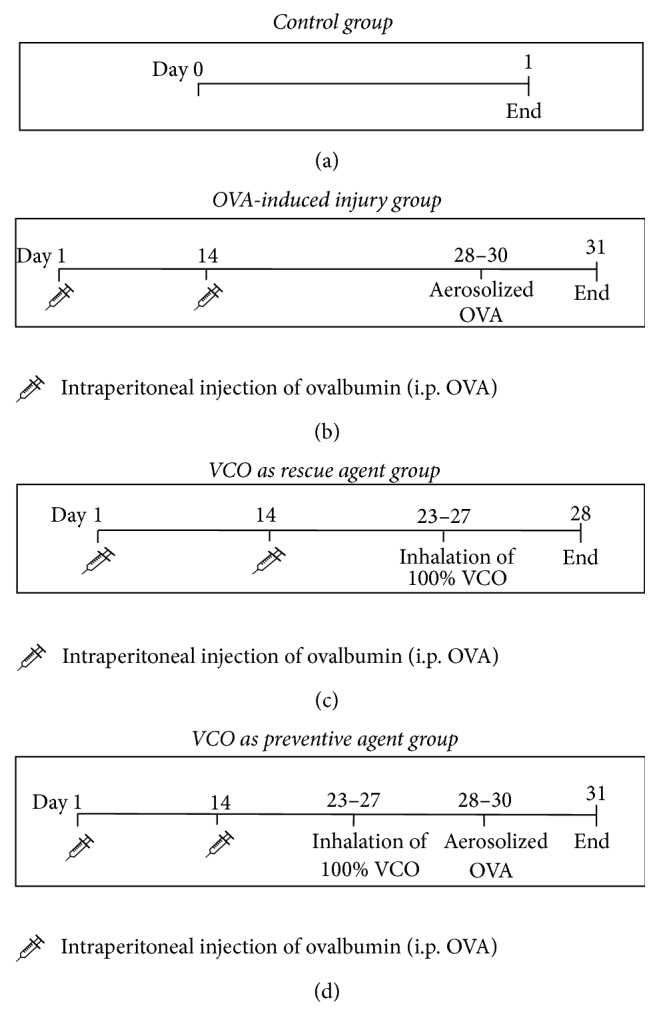
Experimental design used in this study. The control group serve as negative control; meanwhile OVA-induced injury group serve as positive control for lung injury. Treatment groups were the VCO as a rescue agent group and VCO as a preventive agent group. Adapted and modified from Kumar et al. (2008) and Kamaruzaman et al. (2014) [17, 18].

Male New Zealand white rabbits (*n* = 25) weighing 2.60 ± 0.38 kg were used in this study. The study was approved by* The Animal Research Ethics Committee of USM (JEHUSM)* (USM/Animal Ethics Approval/2012/(77)(379)). The rabbits were maintained in a ventilated room and fed daily with water and pellets. The rabbits were divided into five groups (*n* = 5 each): (1) negative control (naïve group), (2) i.p. ovalbumin (OVA) control, (3) OVA inhalation control, (4) VCO as a rescue agent, and (5) VCO as a preventive agent. Groups (2) and (3) were later combined into the OVA-induced injury group.  Figure 1 shows the timeline for treatment in each group.

### 2.3. Sample Collection

Blood, bronchioalveolar lavage (BAL) fluid, and lung were collected for whole blood count (WBC) and inflammatory cell staining. The rabbits were first anesthetised using ketamine (35 mg/kg) and ilium xylazyl (4 mg/kg) via intramuscular injection and blood was collected. Following blood collection, the BAL fluid from each rabbit was collected by lavaging the lung via the trachea with 3 ml of phosphate buffered saline (PBS) twice. The collected BAL fluid was centrifuged at 400 xg for 5 min at 4°C. The supernatant was stored at −70°C for later use. The pellet was used for inflammatory cell staining. The lung collected was according to standard protocol for tissue processing and embedding. The processed tissue blocks were proceeded with embedding process and then sectioned into 5 *μ*m thick slices using a microtome. The tissue ribbons were collected, placed on normal and polysine-coated slides, and air-dried prior to histological analysis.

### 2.4. Histological Analysis

The tissue ribbons on normal and polysine-coated slides as well as cytospun cells from BAL fluid were used for histological analysis. Wright-Giemsa staining, hematoxylin and eosin (H&E) staining, and alcian blue-periodic acid Schiff (AB-PAS) staining were performed following standard staining procedures.

Immunohistochemical (IHC) staining for PCNA marker was conducted following the manufacturer's protocol of the DAKO ARK™ Peroxidase kit (DAKO ARK Peroxidase, Dako, Glostrup, Denmark). In this study, 250 *μ*L of primary antibody cocktail, which contained anti-PCNA antibody (PC10) (proliferation marker, prediluted ab912, Abcam, Cambridge, MA, USA), biotinyl reagent (DAKO ARK Peroxidase), blocking reagent (DAKO ARK Peroxidase), and antibody diluent (DAKO ARK), was used. Streptavidin-HRP (DAKO ARK Peroxidase) was used as the secondary antibody.

### 2.5. Statistical Analysis

All data were expressed as the mean ± standard deviation (SD) of triplicate analyses. Statistical differences among the data collected were determined with two-sided Kolmogorov-Smirnov independent nonparametric tests using SPSS 20. *p* ≤ 0.05 was considered to be statistically significant.

### 2.6. Fatty Acid Content of VCO

*(a) Preparation of Crude Extract*. The extraction of fatty acids from the VCO was carried out using ethanol as the solvent in a 1 : 10 (v/v) sample to solvent ratio. The extract pellets were dissolved in methanol as the solvent carrier to be used in the GC-MS analysis.

*(b) Gas Chromatography-Mass Spectrometry (GC-MS)*. The VCO crude extracts were diluted to 10 mg/ml with ethanol. Quantitative GC-MS analysis was performed using Agilent 5975C GC/MSD, an automated gas chromatograph system (Agilent Technologies, CA, USA). In this analysis, the VCO extracts were introduced by thermal desorption and split injection at 250°C at 10 : 1 into a HP5-MS column with 30 m × 0.25 mm i.d. and film thickness of 0.25 *μ*m. The split flow was maintained at 8 ml/min. The carrier gas, helium, was kept at a constant flow rate of 3 ml/min. The oven temperature program was set as follows: 70°C for 2 min, 5°C/min increase to 250°C for 10 min, and hold at 300°C for 45 min. The temperature of the ionization chamber was set at 250°C, and the ionization stage was performed by electron impact at 70 eV. The detected peaks were identified by comparing the retention time and mass spectra with the mass spectral library in the online mass spectral database from National Institute of Standard and Technology (NIST05). All matches above 0.9 were selected in this study.

## 3. Results

### 3.1. Development of the Allergic Asthma Model

**Figure 2:**
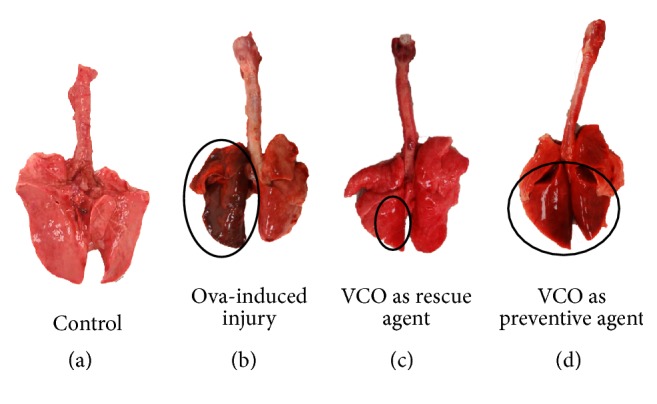
Haemorrhage areas on the sensitized lung, represented by circle. (a) Control; (b) OVA-induced injury; (c) VCO as a rescue agent group; (d) VCO as a preventive agent group. In the OVA-induced injury group (b), the rabbits were injected with OVA together with alum, which acts as an adjuvant; later, some were exposed to repeated inhalation of OVA. Rabbits were given VCO via nasal inhalation for 5 days. The degree of inflammation of the lung (black circle) was observed following the sensitization stage using i.p. OVA and inhalation of OVA. The haemorrhage areas were reduced in both treatment groups (rescue (c) and preventive (d)), especially in the rescue group.

The allergic asthma model was developed by exposing rabbits to OVA with aluminium (alum) hydroxide via i.p. injection and OVA inhalation. In the OVA inhalation group, the i.p. injection was followed by inhalation of OVA. The injury in the lungs was detectable during postmortem as signs of lung haemorrhage, which was observed following allergen sensitization (Figure 2).

**Figure 3:**
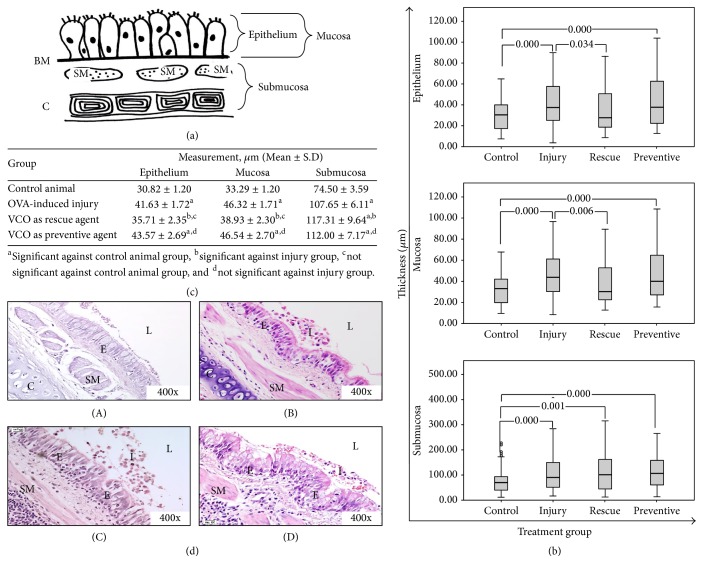
Structural changes of the airway. (a) Illustration of the epithelium, mucosa, and submucosa measurement area for H&E stained slides. The mucosa area ranges from the epithelial cell to the end of the basement membrane (BM). The submucosa area ranges from the smooth muscle (SM) to the end of the cartilage (C). The measurements were in microns (*μ*m). (b) Thickness measurements of airway structures. The boxplot shows the measurements (mean ± SD) made for three parameters: epithelium, mucosa, and submucosa. (c) Morphometric analysis of airway structure of airway lumen. The data were subjected to the two-sided nonparametric analysis using SPSS software. The outliers represent high and low data readings. *p* < 0.05 was considered to be statistically significant. (d) H&E staining for each treatment group. (A) Control, (B) OVA-induced injury, (C) VCO as a rescue agent, and (D) VCO as a preventive agent. Following OVA inhalation, the epithelium (E) was thicker as compared to the control animal. Infiltration of inflammatory cells (I) was also found in the airway lumen (L), indicating that the injury was developed. Following VCO inhalation, airway remodelling (epithelium, mucosa, and submucosa structure) was reduced compared to the OVA-induced injury group.* I, inflammatory cell infiltration; E, epithelium; SM, smooth muscle; C, cartilage; L, airway lumen.*

The development of allergic asthma in the animal model was illustrated by structural changes of the airway (epithelium, mucosa, and submucosa; Figure 3(a)). The observed changes included epithelium thickness and disruption of the epithelial structure, and infiltration of inflammatory cells into the airway lumen (Figure 3(d)(B)). Morphometric analyses have shown significant changes on the airway structures of the injury model as compared to the control group: the thickness of the epithelium (41.6277 ± 1.7245 (*p* = 0.000)), mucosa (46.3162 ± 1.7054 (*p* = 0.000)), and submucosa (107.6487 ± 6.1114 (*p* = 0.000)) in the OVA-induced injury group (Figure 3(c)).

**Figure 4:**
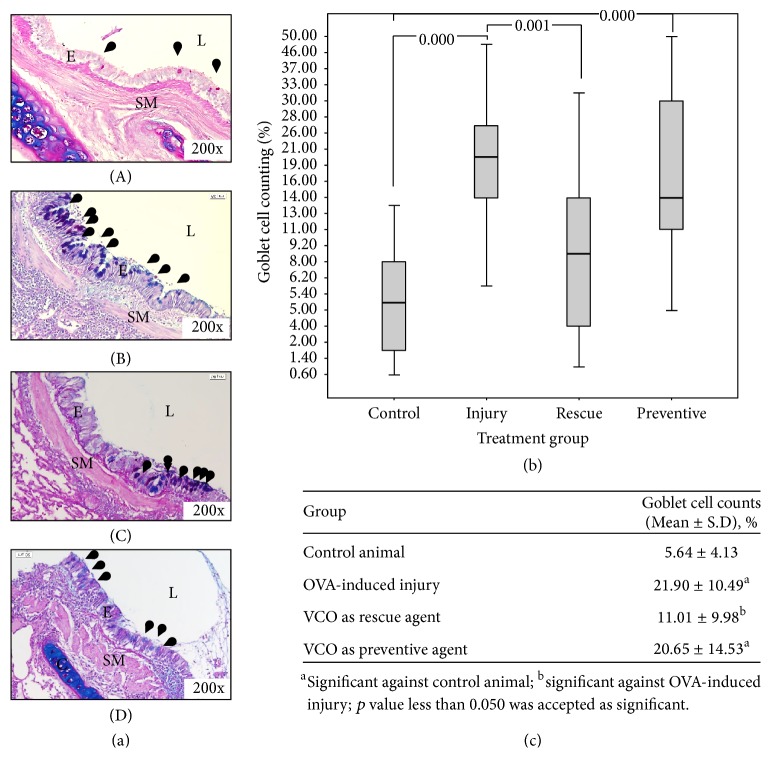
Staining of goblet cells. (a): (A) Control, (B) OVA-induced injury, (C) VCO as a rescue agent, and (D) VCO as preventive agent groups. Goblet cells (black arrow) present in all treatment groups but at different densities. Positive staining for goblet cells are indicated by blue colour for acid mucus, red colour for neutral mucus, and magenta colour for mixed mucus.* C, cartilage; SM, smooth muscle; E, epithelium; L, airway lumen.* (b) Distribution of goblet cells in different treatment groups. The boxplot shows the mean ± SD for each treatment group. Morphometric analysis of the goblet cells in each treatment group revealed significant differences between control animals and the OVA-induced injury group (*p* = 0.000), control animals versus the VCO as a preventive agent group (*p* = 0.000), and OVA-induced injury versus VCO as a rescue agent group (*p* = 0.001). (c) Morphometric analysis of the goblet cell counts. *p* < 0.05 was considered to be statistically significant.

Mucous oversecretion represented by the goblet cell hyperplasia was also observed in the OVA-induced injury group (Figure 4(a)(B)). Following induction using OVA and alum, the numbers of mucous-producing cells, the goblet cells, were significantly increased (*p* < 0.05) in the injury group relative to the control group (Figures 4(b) and 4(c)). Goblet cell hyperplasia, the important feature in asthmatic airways, was well developed in this study (Figure 4(a)(A) versus Figure 4(a)(B)), indicating the development of airway inflammation in this model.

### 3.2. Effect of VCO Inhalation on the Animal Model of Allergic Asthma

During postmortem, the lung haemorrhage was observed in the OVA-induced injury group, VCO as a rescue agent group (Figure 1(c)), and VCO as a preventive agent group (Figure 1(d)). Following injury, the animals were exposed to VCO inhalation as a rescue agent (28 d time point) or a preventive agent (31 d time point). After VCO inhalation, haemorrhage severity in the lungs was reduced in both treatment groups, but better reduction was observed in the rescue group (Figure 2; VCO as rescue agent).

**Figure 5:**
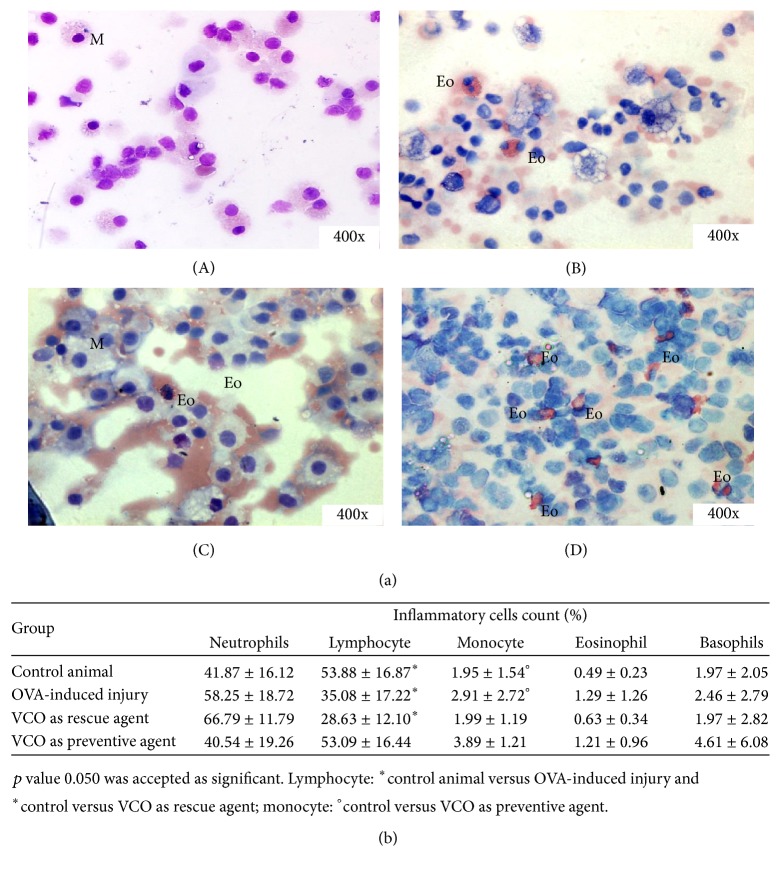
The infiltration of inflammatory cells in different groups. (a) Different types of inflammatory cells were observed in BAL fluid from different treatment groups: (A) Control, (B) OVA-induced injury, (C) VCO as a rescue agent, and (D) VCO as a preventive agent. The distributions of the inflammatory cells differed among groups.* M, mast cell; Eo, eosinophil.* (b) Results of whole blood cell screening. Abundance of different cell types is expressed in percentage (%) of the total cell count. *p* < 0.05 was considered to be statistically significant.

The different types of inflammatory cells were identified by different levels of cytoplasmic granularity and the morphology of the nucleus. The total cell count (expressed as percentage of cells) showed that the numbers of proinflammatory cells (eosinophils and basophils) were high in the OVA-induced injury group (1.29% ± 1.26% and 2.46% ± 2.79%, resp., Figure 4(b)), indicating that injury had developed in the animal model. Treatment with VCO did not significantly reduce the total cell count for proinflammatory cells, but the mean values for eosinophils and basophils were reduced in the VCO as a rescue agent group (0.63 ± 0.34 and 1.97 ± 2.82, resp.) but not in the VCO as a preventive agent group (1.21 ± 0.96 and 4.61 ± 6.08, resp.). In the VCO as a rescue group, the neutrophil count was increased (66.79 ± 11.79) but the lymphocyte count was decreased (28.63 ± 12.10) relative to the OVA-induced injury group. However, the monocyte count for the rescue group was comparable to that of the control group. Although differences were observed for cell count between each treatment group, the differences were not statistically significant except for lymphocytes and monocytes (Figure 5(b)).

H&E stained lung sections showed structural differences between the injury (Figure 3(d)(B)) and treatment groups (Figure 3(d)(C and D)) compared to control animals (Figure 3(d)(A)). In the OVA-induced injury group (Figure 3(d)(B)), the arrangement of the epithelium and the structure of the mucosa layer were disrupted, and infiltration of inflammatory cells into the airway lumen was observed. This observation supported the findings of the morphometric analysis, which showed significant differences (*p* < 0.05) in all structures between injury and control animals.

After treatment with VCO, the morphometric analysis revealed significant differences between the injury and the VCO as a rescue group for epithelium thickness (35.71 *μ*m ± 2.35 *μ*m, *p* = 0.034) and mucosa thickness (38.93 *μ*m ± 2.30 *μ*m, *p* = 0.006) but not for submucosa thickness (117.31 *μ*m ± 9.64 *μ*m, *p* = 0.689). However, no significant differences for any parameter measured were detected between the injury and the VCO as a preventive agent group (Figure 3(b)).

AB-PAS staining revealed that goblet cells were abundant in the OVA-induced injury group (21.90 ± 10.49%) (Figure 4(a)(B)) compared to the control group (5.64 ± 4.13%) (Figure 4(a)(A)). After inhalation of VCO, the goblet cells were sparsely observed in the VCO as a rescue agent group (11.01 ± 9.98%). However, the goblet cell count in the VCO as a preventive agent group (20.65 ± 14.53%) was similar to that of the OVA-induced injury group. Overall, significant differences were observed between the control and OVA-induced injury groups (*p* = 0.000), the control and VCO as a preventive agent group (*p* = 0.000), and the OVA-induced injury and VCO as a rescue agent group (*p* = 0.000). The number of goblet cells did not differ significantly between the VCO as a rescue agent group and the control group (Figure 4(c)).

**Figure 6:**
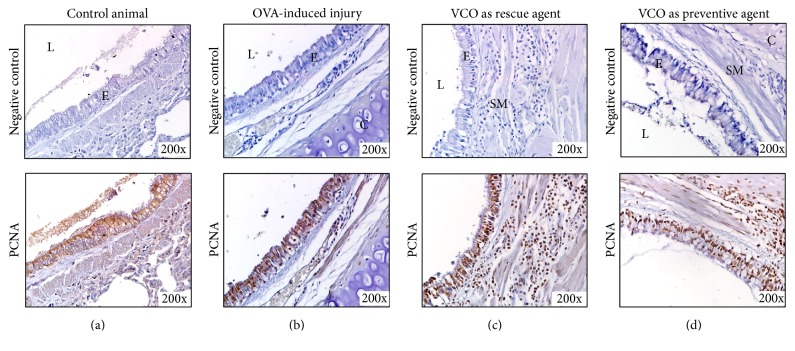
Distribution of PCNA-stained cells in all treatment groups. The actively proliferating cells are stained with PCNA. Positively stained cells can be identified by the deep brown nuclei. In control animals (a), positively stained cells are infrequent among the alveolar cells. Exposure to the allergen (OVA and alum) increased the number of positively stained cells in the injury group (b), indicating that the cells are actively dividing and proliferating to combat infiltration of the sensitizer into the lung. The cells are observed at the basal area of the epithelium (E), smooth muscle (SM), and alveolar cells. After treatment with VCO, positively stained cells are also observed among the airway epithelial cells and cell infiltrates in the submucosa area (c and d). C, cartilage.

In this study, proliferation of the airway cells was observed during the repair process. Positively PCNA-stained cells were infrequently found in the control animal tissue (Figure 6(a), lower panel), and those present were mostly found among alveolar cells. After exposure to the injury agent, proliferative cells become abundant, indicating that the cells were actively dividing and proliferating in order to combat infiltration of the sensitizer (i.e., OVA). The cells were observed at the basal area of the epithelium and smooth muscle and among alveolar cells (Figure 6(b), lower panel). After treatment with VCO, the positive-stained cells were distributed among the airway epithelial and cell infiltrates in the submucosa area (Figures 6(c) and 6(d), lower panel). However, the number of proliferative cells in the VCO as a rescue agent group was lower among the cell infiltrates and alveolar area compared to that of the VCO as a preventive agent group (Figure 6(c), lower panel).

### 3.3. Fatty Acid Content of VCO

GC-MS analysis revealed the presence of 17 compounds in the VCO extract. Most of the volatile compounds detected consisted of free fatty acids, methyl groups, benzene groups, and traces of metals. The dominant peak (total peak area ~ 40.86%) was a 12-carbon compound known as dodecanoic acid (i.e., lauric acid) (Table 1). The percentage of the peak area (% area) represents the percentage of a given compound among the total compounds in the VCO sample.

## 4. Discussion

In animal models of chronic lung injury, ovalbumin, which is a protein from chicken eggs, frequently is used as an allergen. This substance can induce robust, allergic pulmonary inflammation in laboratory rodents [19]. In studies focused on the action of inflammatory cells, OVA with the addition of alum was used to elicit an immune response, thereby mimicking airway inflammation condition in humans [6, 20–23]. During the sensitization stage, alum acts as an adjuvant that can enhance the action of OVA against the defence systems of the body [21, 23–26]. The addition of alum during injection of OVA not only induces but also increases the production of inflammatory cells, especially Th_2_, Th_1_, IL4, and IL5 [25]. The use of alum in inducing chronic lung injury, especially asthma, has been well established and widely used in rat, mouse, and rabbit models [24, 26–28].

In the current study, the injured area of the lung was detected by the presence of haemorrhage, which was caused mainly by repeated exposure to the allergen (OVA and alum). Severe haemorrhage was observed in lung samples from the OVA-induced injury group. This condition was reflected by high infiltration of inflammatory cells in the BAL fluid. Eosinophils are the most prominent inflammatory cells in asthma, and they may be a mediator for the epithelial damage that occurs during asthma pathogenesis [29, 30]. Other common features of airway injury in asthma are remodelling of the airway structures, which includes epithelial damage, increased airway smooth muscle, mucous gland hypertrophy, and goblet cell hyperplasia [31–34]. These injury features limit airway function by thickening the airway wall and narrowing the airway lumen, thus reducing the airway flow rate; these processes most likely are due to eosinophilic and neutrophilic infiltration across the alveolar wall into the alveolar spaces [35–38]. Thickening of the airway smooth muscle, inflammatory cell infiltration into the alveolar spaces and lamina propria layer, epithelial hypertrophy, and goblet cell hyperplasia were all observed in the OVA-induced injury group but not in the control group (Figures 3, 4, and 5). This cellular response proved that the rabbit model for an allergic airway inflammation was well developed prior to treatment with VCO inhalation, at least for some features that mimic human condition of asthma.

The main objective of this study was to evaluate the anti-inflammatory effects of VCO in reducing asthma-related features (i.e., infiltration of inflammatory cells, remodelling of airway structure, and goblet cell hyperplasia). The effect of VCO inhalation was measured by its ability to eliminate the inflammatory cells, including eosinophils, which are known to play an important role in the pathogenesis of airway disorders [39]. Our findings showed that VCO inhalation was effective at alleviating the inflammatory responses in the airway, but the response was more profound in the rescue group than in the preventive group. Anti-inflammatory effect of the VCO has been reported by Intahphuak et al. (2010) where, in acute inflammatory models of ethyl phenylpropiolate-induced ear edema in rat, the VCO treatment gave moderate anti-inflammatory effect. The VCO was also found to be able to reduce the transudative weight, granuloma formation, and serum ALP activity [13]. Another finding on anti-inflammatory effect of the VCO also has been recorded by Zakaria et al. (2011). The study stated that the VCOs exhibited anti-inflammatory activity in an acute (carrageenan-induced paw edema test), but not in a chronic (cotton-pellet-induced granuloma test) model of inflammation [40]. In both groups, the number of eosinophils was reduced relative to the OVA-induced injury group, and the reduction pattern was similar for other types of inflammatory cells (i.e., lymphocytes, monocytes, and basophils). Reduction in the inflammatory cell counts was also reflected by the structural changes of the airway, as the infiltration of inflammatory cells led to remodelling of airway structures (epithelium, mucosa, and submucosa). In the VCO as a rescue agent group, epithelium and mucosa thickness was significantly reduced compared to that of the OVA-induced injury group. However, these measurements did not differ significantly between the preventive group and the OVA-induced injury group, which might be due to the repeated inhalation of OVA after VCO inhalation (days 28, 29, and 30) that might have stimulated continuous infiltration of inflammatory cells.

In the healthy airway, the main function of mucin is to maintain the sterility of the lower airspace by trapping foreign particles in the upper airways [41]. The secretion of mucin is also crucial for humidifying the alveolar surface for optimum gaseous exchange in the lung. However, overproduction of mucin (or mucous hypersecretion) due to goblet cell hyperplasia triggered by the infiltration of pathogens into the lung spaces leads to increased severity of asthma. The model of allergic airway in the current study was successfully developed, in which some features mimic human conditions of asthma, as indicated by mucous overproduction due to goblet cell hyperplasia in the OVA-induced injury group. Extensive studies conducted by several researchers have documented direct involvement of goblet cell hyperplasia in the severity of asthma pathogenesis [34, 42–44]. Ordoñez et al. (2001) reported that the chronic narrowing of the airway lumen in moderate and mild asthma was mainly caused by the accumulation of mucus along the airway [44]. In addition, Dong et al. (2012) reported that activation of goblet cell hyperplasia is closely related to infiltration of eosinophils into the airway lumen during the sensitization stage [45]. Inhalation of VCO resulted in a significant reduction of goblet cells hyperplasia, especially in the VCO as a rescue agent group. This might be due to the action of anti-inflammatory effects of VCO that facilitates in reducing the severity of the mucous secretion after allergen sensitization. Thus, this finding indicates that, by reducing the inflammatory cell responses due to ova induction, the inhalation of VCO also reduced the goblet cell hyperplasia (i.e., mucous overproduction) in this model of airway inflammation. Behind the pathogenesis of the mucus overproduction and goblet cell hyperplasia, most prominent genes that contribute to these asthma characteristics are the A Disintegrin And Metalloproteinases (ADAMs) and ADAM with Thrombospondin (ADAMTS), and the aquaporin family [45–47]. A significant regulation of this gene might be one of the possible answers for the effectiveness of our treatment on animal model. Further study to elucidate the molecular regulation of mucus-secreting related genes is important as to determine the mechanisms on how the VCO controls the overproduction of mucus and goblet cell hyperplasia in a model of allergic asthma. The genes that can be evaluated further are Th2-related genes (interleukin 4 (IL-4), interleukin-5 (IL-5), and interleukin-13 (IL-13)), transforming growth factor-*β* (TGF-*β*), mucin (MUC) genes (MUC5AC, MUC5B), Signal Transducer and Activator of Transcription 6 (STAT6), and forkhead box A2 (FOXA2) which are closely involved in asthma onset and progression.

The infiltration of active inflammatory cells during an allergen attack in the lungs leads to hypertrophy of smooth muscle cells and epithelial cells, which subsequently leads to airway remodelling, which in turn narrows the airway lumen [4, 48, 49]. Increase in the number of proliferation activity of the airway epithelial cells in response to allergen induction to the airway was supported by the high numbers of PCNA-stained cells in the OVA-induced injury group. Following treatment with VCO, fewer PCNA-positive cells were present in the VCO in a rescue group but not in the VCO as a preventive group. This finding correlates with the infiltration of inflammatory cells (Figure 5(b)) and airway structure remodelling (Figures 3(c) and 3(d)) results where fewer proinflammatory cells were found and reduction in epithelium and mucosa thicknesses in the rescue group (Figure 3(c)). The difference between the rescue and preventive agent groups (i.e., a series of repeated inhalations of OVA before the samples were collected) explains this result. Repeated inhalation of an allergen increases the infiltration of inflammatory cells into the area of injury, thus leading to greater severity of asthma pathogenesis. The involvement of inflammatory cells during injury activates cell proliferation, which is required to repair the injury. Activation of PCNA is associated with active replication and reparation of DNA during the cell cycle [50–52].

Repeated inhalation of OVA following treatment with VCO in the preventive group was used to determine if VCO can be used to replace long-acting-beta-2 agonists (LABAs, preventive drugs) or only to replace short-acting-beta-2 agonists (SABAs, rescue drug) in current asthma treatment. We found that reexposure to the allergen (OVA) in the preventive group increased the infiltration of proinflammatory cells (eosinophils and basophils) into the area of injury (Figure 5(b)). We believe that this was the main reason why VCO acted better as a rescue agent than a preventive agent.

Several drugs are available for quick relief during asthma attacks. However, due to the long-term side effects of prolonged usage of these drugs, natural products are becoming the focus of studies designed to discover complementary and alternative treatments. Natural products are well known for their high antioxidant content and their ability to replace conventional drugs for therapy. Extracting VCO from mature fresh coconut meat can be performed using wet or dry methods [12, 53]. In this study, a wet method was employed, which involved chilling and thawing processes. Both low (5°C) and high (25°C) temperature were used to destabilize the coconut milk emulsion in order to extract the oil from the oil globules in the chilled coconut milk [54]. Previous studies evaluated different methods for production of VCO and reported that the most favourable methods for producing VCO with high fatty acid and oil recovery were the chilling and thawing method and the enzymatic method [54–56].

Since our interest was to know what might be the main compounds contributing to the anti-inflammatory effects of VCO inhalation, the VCO was then screened for possible antioxidant, anti-inflammatory, and antiasthmatic components using GC-MS technology. GC-MS analysis revealed the presence of 17 volatile organic compounds from the methanol extracts of the VCO. Lauric acid was the main medium-chain triglyceride present in the VCO, and it represented 40.86% of the total content. The Asian and Pacific Coconut Community (APCC) standard for lauric acid content of VCO is 45–56% [56–58], which is slightly higher than the value for VCO in our study. It is important to note that the content of active compounds varies between extraction and source of VCO. The fatty acid content of VCO is strongly affected by the temperature and humidity of the working environment during production and by the source of the coconut; thus the differences between our sample and the standard might be due to external conditions such as source of the coconut, mechanical forces, temperature during the production stage, and the storage conditions of the VCO itself [59]. The first and third most prevalent fatty acids (lauric acid and capric acid, resp.) might be responsible for the effectiveness of VCO treatment via the inhalation route as a rescue agent, as they are known to have immunosuppression properties. Further study to evaluate the effect of prominent active compounds of VCO on allergic asthma model should be carried out.

Several studies reported that lauric acid has high antiviral properties against lipid-coated RNA and DNA viruses, antimicrobial activity against pathogenic Gram negative and positive bacteria, antiprotozoal activity, and antifungal activity [60–62]. Capric acid (decanoic acid) also contributes to the beneficial effect of VCO. It is mainly found in tropical oils such as coconut oil, and the standard proportion in VCO ranges from 4 to 8%. In our analysis it represented 5.17% of the fatty acid content of the VCO. Capric acid and lauric acid together represent powerful antioxidant and antimicrobial components of coconut oil [60, 63]. Free fatty acids available in this natural oil have been studied for their beneficial effects on many aspects of health diseases. Various studies have been done by scientists worldwide with some reporting that VCO exhibits antibacteria [11], antiviruses, anti-HIV, anti-inflammatory [40], and antidiabetes properties [16, 64] and has been extensively used as topical application in treating skin disorders [15, 65]. Study by Nurul-Iman et al. (2013) showed that VCO is able to prevent blood pressure elevation and improves endothelial function in rats [66]. The efficacy and safety of the virgin coconut oil in reducing visceral adiposity also have proved that VCO is efficacious as weight reducing and safe for human consumption [16].

## 5. Conclusion

This study showed that VCO inhalation can alleviate inflammatory conditions of the airways following i.p. OVA treatment and OVA inhalation in a rabbit model of allergic asthma. The asthma features observed in this study were found to mimic the human conditions of asthma. Our interest was to evaluate the effectiveness of the VCO whether to act as a rescue and/or a preventive agent in reducing symptoms of allergic asthma. Our current study revealed that the VCO inhalation reduced infiltration of proinflammatory cells, especially eosinophils, into the airway cavity of the alveolar spaces, which might be later used as an alternative treatment for managing asthma conditions where the treatment was found more effective as a rescue agent than as a preventive agent. This might be due to the experimental design used for the preventive group, in which rabbits were reexposed to OVA via inhalation on days 28, 29, and 30 before samples were collected. Lauric acid and capric acid were abundant in our VCO sample, and they are known to have anti-inflammatory properties. The combination of these fatty acids in the VCO may explain the effectiveness of VCO inhalation in reducing asthma-related symptoms. However, further study should be carried out in order to confirm which specific compounds are present in the VCO that contributes to the anti-inflammatory effects as well as the possible molecular target which can be served as specific treatment platform in the future. The clinical study should be carefully planned in order to confirm the effectiveness of VCO in treatment of patients. The current study suggests the VCO inhalation might be a potential complementary and alternative medication in managing asthma patients.

## Figures and Tables

**Table 1:** List of free fatty acids detected in the VCO sample.

IUPAC	Common name	Lipid number	Molecular formula	Percentage of composition (%)
Dodecanoic acid	Lauric acid	C12:0	C_12_H_24_O_2_	40.86
Methyl tetradecanoate	Methyl myristate	C15:0	C_15_H_30_O_2_	11.14
Octanoic acid	Caprylic acid	C8:0	C_18_H_16_O_2_	7.69
Decanoic acid	Capric acid	C10:0	C_10_H_20_O_2_	5.17
Hexadecanoic acid	Palmitic acid	C16:0	C_16_H_32_O_2_	4.22
9-Octadecenoic acid	Oleic acid	C18:1 n-9	C_18_H_34_O_2_	0.33

The IUPAC nomenclature was referred as International Union of Pure and Applied Chemistry. All the compounds listed were selected based on the 0.9 matching with the National Institute of Standard and Technology (NIST) database.

## References

[1] Barnes P. J. (2008). Immunology of asthma and chronic obstructive pulmonary disease. *Nature Reviews Immunology*.

[2] Keir S., Page C. (2008). The rabbit as a model to study asthma and other lung diseases. *Pulmonary Pharmacology & Therapeutics*.

[3] Hernandez M. L., Herbst M., Lay J. C. (2012). Atopic asthmatic patients have reduced airway inflammatory cell recruitment after inhaled endotoxin challenge compared with healthy volunteers. *Journal of Allergy and Clinical Immunology*.

[4] Davies D. E., Wicks J., Powell R. M., Puddicombe S. M., Holgate S. T. (2003). Airway remodeling in asthma: new insights. *Journal of Allergy and Clinical Immunology*.

[5] El Gazzar M., El Mezayen R., Marecki J. C., Nicolls M. R., Canastar A., Dreskin S. C. (2006). Anti-inflammatory effect of thymoquinone in a mouse model of allergic lung inflammation. *International Immunopharmacology*.

[6] Jung W.-K., Choi I., Oh S. (2009). Anti-asthmatic effect of marine red alga (Laurencia undulata) polyphenolic extracts in a murine model of asthma. *Food and Chemical Toxicology*.

[7] Ra J., Lee S., Kim H. J., Jang Y. P., Ahn H., Kim J. (2010). Bambusae Caulis in Taeniam extract reduces ovalbumin-induced airway inflammation and T helper 2 responses in mice. *Journal of Ethnopharmacology*.

[8] Kim I. S., Yang E. J., Lee J.-S., Yun C.-Y., Ryang Y. S., Kim J.-B. (2011). Suppression of ovalbumin-induced airway inflammatory responses in a mouse model of asthma by Mimosa pudica extract. *Phytotherapy Research*.

[9] Karaman M., Firinci F., Cilaker S. (2012). Anti-inflammatory effects of curcumin in a murine model of chronic asthma. *Allergologia et Immunopathologia*.

[10] Yang E. J., Lee J.-S., Yun C.-Y. (2008). Inhibitory effects of *Duchesnea chrysantha* extract on ovalbumin-induced lung inflammation in a mouse model of asthma. *Journal of Ethnopharmacology*.

[11] Nevin K. G., Rajamohan T. (2010). Effect of topical application of virgin coconut oil on skin components and antioxidant status during dermal wound healing in young rats. *Skin Pharmacology and Physiology*.

[12] Zakaria Z. A., Rofiee M. S., Somchit M. N. (2011). Hepatoprotective activity of dried- and fermented-processed virgin coconut oil. *Evidence-Based Complementary and Alternative Medicine*.

[13] Intahphuak S., Khonsung P., Panthong A. (2010). Anti-inflammatory, analgesic, and antipyretic activities of virgin coconut oil. *Pharmaceutical Biology*.

[14] Kamalaldin N., Sulaiman S., Yahaya B. (2015). Apoptosis in lung cancer cells induced by exposure to virgin coconut oil. *Regenerative Research*.

[15] Verallo-Rowell V. M., Dillague K. M., Syah-Tjundawan B. S. (2008). Novel antibacterial and emollient effects of coconut and virgin olive oils in adult atopic dermatitis. *Dermatitis: Contact, Atopic, Occupational, Drug*.

[16] Liau K. M., Lee Y. Y., Chen C. K., Rasool A. H. (2011). An open-label pilot study to assess the efficacy and safety of virgin coconut oil in reducing visceral adiposity. *ISRN Pharmacology*.

[17] Kumar R. K., Herbert C., Foster P. S. (2008). The "classical" ovalbumin challenge model of asthma in mice. *Current Drug Targets*.

[18] Kamaruzaman N. A., Sulaiman S. A., Kaur G., Yahaya B. (2014). Inhalation of honey reduces airway inflammation and histopathological changes in a rabbit model of ovalbumin-induced chronic asthma. *BMC Complementary and Alternative Medicine*.

[19] Nials A. T., Uddin S. (2008). Mouse models of allergic asthma: acute and chronic allergen challenge. *Disease Models and Mechanisms*.

[20] Nadel J., Takeyama K., Agustí C. (1999). Role of neutrophil elastase in hypersecretion in asthma. *European Respiratory Journal*.

[21] Zosky G. R., Sly P. D. (2007). Animal models of asthma. *Clinical and Experimental Allergy*.

[22] Bates J. H., Rincon M., Irvin C. G. (2009). Animal models of asthma. *AJP: Lung Cellular and Molecular Physiology*.

[23] Kamaruzaman N. A., Kardia E., Kamaldin N., Latahir A. Z., Yahaya B. H. (2013). The rabbit as a model for studying lung disease and stem cell therapy. *BioMed Research International*.

[24] Brewer J. M., Conacher M., Hunter C. A., Mohrs M., Brombacher F., Alexander J. (1999). Aluminium hydroxide adjuvant initiates strong antigen-specific Th2 responses in the absence of IL-4- or IL-13-mediated signaling. *The Journal of Immunology*.

[25] Pichavant M., Goya S., Hamelmann E., Gelfand E. W., Umetsu D. T. (2007). Unit 15.18 Animal models of airway sensitization. *Current Protocols in Immunology*.

[26] Conrad M. L., Yildirim A. Ö., Sonar S. S. (2009). Comparison of adjuvant and adjuvant-free murine experimental asthma models. *Clinical and Experimental Allergy*.

[27] Chung S. H., Choi S. H., a Choi J., Chuck R. S., Joo C. K. (2012). Curcumin suppresses ovalbumin-induced allergic conjunctivitis. *Molecular Vision*.

[28] Banerjee E. R., Henderson W. R. (2012). Characterization of lung stem cell niches in a mouse model of bleomycin-induced fibrosis. *Stem Cell Research and Therapy*.

[29] Wardlaw A. J., Brightling C., Green R., Woltmann G., Pavord I. (2000). Eosinophils in asthma and other allergic diseases. *British Medical Bulletin*.

[30] Bradding P. (2008). Asthma: eosinophil disease, mast cell disease, or both?. *Allergy, Asthma & Clinical Immunology*.

[31] Saetta M., Turato G. (2001). Airway pathology in asthma. *European Respiratory Journal*.

[32] Toward T. J., Broadley K. J. (2002). Goblet cell hyperplasia, airway function, and leukocyte infiltration after chronic lipopolysaccharide exposure in conscious guinea pigs: effects of rolipram and dexamethasone. *Journal of Pharmacology and Experimental Therapeutics*.

[33] Aoshiba K., Nagai A. (2004). Differences in airway remodeling between asthma and chronic obstructive pulmonary disease. *Clinical Reviews in Allergy and Immunology*.

[34] Park K.-S., Korfhagen T. R., Bruno M. D. (2007). SPDEF regulates goblet cell hyperplasia in the airway epithelium. *Journal of Clinical Investigation*.

[35] Choe M. M., Sporn P. H., Swartz M. A. (2003). An in vitro airway wall model of remodeling. *American Journal of Physiology - Lung Cellular and Molecular Physiology*.

[36] Balzar S., Chu H. W., Silkoff P. (2005). Increased TGF-*β*2 in severe asthma with eosinophilia. *Journal of Allergy and Clinical Immunology*.

[37] Chen G., Wan H., Luo F. (2010). Foxa2 programs Th2 cell-mediated innate immunity in the developing lung. *Journal of Immunology*.

[38] Hallstrand T. S., Woodruff P. G., Holgate S. T., Knight D. A. (2012). Function of the airway epithelium in asthma. *Journal of Allergy*.

[39] Bhattacharyya S., Zhao Y., Kay T. W. H., Muglia L. J. (2011). Glucocorticoids target suppressor of cytokine signaling 1 (SOCS1) and type 1 interferons to regulate Toll-like receptor-induced STAT1 activation. *Proceedings of the National Academy of Sciences of the United States of America*.

[40] Zakaria Z. A., Somchit M. N., Mat Jais A. M., Teh L. K., Salleh M. Z., Long K. (2011). In vivo antinociceptive and anti-inflammatory activities of dried and fermented processed virgin coconut oil. *Medical Principles and Practice*.

[41] Thai P., Loukoianov A., Wachi S., Wu R. (2008). Regulation of airway mucin gene expression. *Annual Review of Physiology*.

[42] Curran D. R., Cohn L. (2010). Advances in mucous cell metaplasia: A plug for mucus as a therapeutic focus in chronic airway disease. *American Journal of Respiratory Cell and Molecular Biology*.

[43] Lumsden A. B., Mclean A., Lamb D. (1984). Goblet and Clara cells of human distal airways: evidence for smoking induced changes in their numbers. *Thorax*.

[44] Ordoñez C. L., Khashayar R., Wong H. H. (2001). Mild and moderate asthma is associated with airway goblet cell hyperplasia and abnormalities in mucin gene expression. *American Journal of Respiratory and Critical Care Medicine*.

[45] Dong C., Wang G., Li B. (2012). Anti-asthmatic agents alleviate pulmonary edema by upregulating AQP1 and AQP5 expression in the lungs of mice with OVA-induced asthma. *Respiratory Physiology and Neurobiology*.

[46] Paulissen G., Rocks N., Gueders M. M. (2009). Role of ADAM and ADAMTS metalloproteinases in airway diseases. *Respiratory Research*.

[47] Shen Y., Wang Y., Chen Z. (2011). Role of aquaporin 5 in antigen-induced airway inflammation and mucous hyperproduction in mice. *Journal of Cellular and Molecular Medicine*.

[48] Sterk P. (1992). The determinants of the severity of acute airway narrowing in asthma and COPD. *Respiratory Medicine*.

[49] Halwani R., Al-Muhsen S., Al-Jahdali H., Hamid Q. (2011). Role of transforming growth factor-*β* in airway remodeling in asthma. *American Journal of Respiratory Cell and Molecular Biology*.

[50] Citterio S., Sgorbati S., Levi M., Colombo B. M., Sparvoli E. (1992). PCNA and total nuclear protein content as markers of cell proliferation in pea tissue. *Journal of Cell Science*.

[51] Kubben F. J. G. M., Peeters-Haesevoets A., Engels L. G. J. B. (1994). Proliferating cell nuclear antigen (PCNA): a new marker to study human colonic cell proliferation. *Gut*.

[52] Koundrioukoff S., Jónsson Z. O., Hasan S. (2000). A direct interaction between proliferating cell nuclear antigen (PCNA) and Cdk2 targets PCNA-interacting proteins for phosphorylation. *Journal of Biological Chemistry*.

[53] Abujazia M. A., Muhammad N., Shuid A. N., Soelaiman I. N. (2012). The effects of virgin coconut oil on bone oxidative status in ovariectomised rat. *Evidence-Based Complementary and Alternative Medicine*.

[54] Mansor T. S. T., Che Man Y. B., Shuhaimi M., Abdul Afiq M. J., Ku Nurul F. K. M. (2012). Physicochemical properties of virgin coconut oil extracted from different processing methods. *International Food Research Journal*.

[55] Bawalan D. D. Processing manual for virgin coconut oil, its products and by-products for pacific island countries and territories, 2011.

[56] Marina A. M., Man Y. B., Amin I. (2009). Virgin coconut oil: emerging functional food oil. *Trends in Food Science & Technology*.

[57] Dayrit F. M., Erin Buenafe O. M., Chainani E. T. (2007). Standards for essential composition and quality factors of commercial virgin coconut oil and its differentiation from RBD coconut oil and copra oil. *Philippine Journal of Science*.

[58] Elfianus G. (2008). Teknik pengolahan Virgin Coconut Oil menggunakan ragi tape. *Buletin Teknologi Pertanian*.

[59] Rohman A., Che Man Y. B., Ismail A., Hashim P. (2011). Monitoring the oxidative stability of virgin coconut oil during oven test using chemical indexes and FTIR spectroscopy. *International Food Research Journal*.

[60] DebMandal M., Mandal S. (2011). Coconut (*Cocos nucifera* L.: Arecaceae): in health promotion and disease prevention. *Asian Pacific Journal of Tropical Medicine*.

[61] Winarsi H., Purwanto A. (2008). Virgin coconut oil ( VCO ) enriched with Zn as immunostimulator for vaginal candidiasis patient. *HAYATI Journal of Biosciences*.

[62] Arunima S., Rajamohan T. (2012). Virgin coconut oil improves hepatic lipid metabolism in rats--compared with copra oil, olive oil and sunflower oil. *Indian Journal of Experimental Biology*.

[63] Ogbolu D. O., Oni A. A., Daini O. A., Oloko A. P. (2007). In vitro antimicrobial properties of coconut oil on Candida species in Ibadan, Nigeria. *Journal of Medicinal Food*.

[64] Nevin K. G., Rajamohan T. (2004). Beneficial effects of virgin coconut oil on lipid parameters and in vitro LDL oxidation. *Clinical Biochemistry*.

[65] Nevin K. G., Rajamohan T. (2009). Wet and dry extraction of coconut oil: Impact on lipid metabolic and antioxidant status in cholesterol coadministered rats. *Canadian Journal of Physiology and Pharmacology*.

[66] Nurul-Iman B. S., Kamisah Y., Jaarin K., Qodriyah H. M. S. (2013). Virgin coconut oil prevents blood pressure elevation and improves endothelial functions in rats fed with repeatedly heated palm oil. *Evidence-Based Complementary and Alternative Medicine*.

